# The chronically ill in the labour market – are they hierarchically sorted by education?

**DOI:** 10.1186/s12939-024-02148-w

**Published:** 2024-03-25

**Authors:** M. Kamrul Islam, Egil Kjerstad, Håvard Thorsen Rydland

**Affiliations:** NORCE Health and Society, Nygårdsgaten 112, Bergen, 5008 Norway

**Keywords:** Chronic disease, Employment probability, Socio-economic status, Education, Shielding effect, Longitudinal data, Norway

## Abstract

**Background:**

The chronically ill as a group has on average lower probability of employment compared to the general population, a situation that has persisted over time in many countries. Previous studies have shown that the prevalence of chronic diseases is higher among those with lower levels of education. We aim to quantify the double burden of low education and chronic illness comparing the differential probabilities of employment between the chronically ill with lower, medium, and high levels of education and how their employment rates develop over time.

**Methods:**

Using merged Norwegian administrative data over a 11-year period (2008–2018), our estimations are based on multivariable regression with labour market and time fixed effects. To reduce bias due to patients’ heterogeneity, we included a series of covariates that may influence the association between labour market participation and level of education. To explicitly explore the ‘shielding effect’ of education over time, the models include the interaction effects between chronic illness and level of education and year.

**Results:**

The employment probabilities are highest for the high educated and lowest for chronically ill individuals with lower education, as expected. The differences between educational groups are changing over time, though, driven by a revealing development among the lower-educated chronically ill. That group has a significant reduction in employment probabilities both in absolute terms and relative to the other groups. The mean predicted employment probabilities for the high educated chronic patient is not changing over time indicating that the high educated as a group is able to maintain labour market participation over time. Additionally, we find remarkable differences in employment probabilities depending on diagnoses.

**Conclusion:**

For the chronically ill as a group, a high level of education seems to “shield” against labour market consequences. The magnitude of the shielding effect is increasing over time leaving chronically ill individuals with lower education behind. However, the shielding effect varies in size between types of chronic diseases. While musculoskeletal, cardiovascular and partly cancer patients are “sorted” hierarchically according to level of education, diabetes, respiratory and mental patients are not.

**Supplementary Information:**

The online version contains supplementary material available at 10.1186/s12939-024-02148-w.

## Introduction

Education is seen as a vehicle of social equality, a mechanism for social mobility. Manifested by the social gradient in health, higher education improves chances of enjoying good health and a longer life. Consequently, people with different levels of education are hierarchically sorted. In absolute terms, those at the bottom layers of the educational hierarchy have on average lower income, weaker employment prospects, and higher mortality. Not the least, they are more likely to develop chronic diseases (non-communicable diseases) compared to those higher in the hierarchy (see e.g., [1–5]).

The chronically ill as a group has on average lower probability of employment and higher share of part time work compared to the general population, a situation that has persisted over time. Tackling chronic illnesses will be critical as the labour force ages [6]. In times of increasing inequality in many jurisdictions, it is discouraging that people with chronic health conditions do not seem to make progress in labour market attachment despite political and administrative efforts to improve the situation.

In Norway, a country characterized by high overall employment, an inclusive working life, and active labour market policies, objectives related to groups with chronic illness and their labour market affiliation have been expressed since the 1960s and reinforced by various governments since then. The main policy measure developed to favour the employment situation and working conditions of people with chronic diseases is the tripartite agreement on a more inclusive working life (IWL).^1^

While previous studies have shown that the prevalence of chronic diseases is higher among those with low(er) level of education, higher education is of course neither a guarantee against deterioration of health, e.g., the onset of chronic health conditions like certain types of cardiovascular diseases, stroke, diabetes, mental health, musculoskeletal and respiratory diseases, nor an insurance against potential negative labour market consequences following the onset of chronic health conditions. Thus, considering the social gradient in health, we aim to quantify the double burden of low education and chronic illness, and study how trends develop over time. Are the higher educated chronically ill relatively more “shielded” against unfortunate labour market consequences? We are concerned that the observed development in the labour market participation among the chronically ill in Norway^2^ is concealing a reproduction of inequality that should alarm policymakers.

The ‘shielding’ effect of education has been studied previously but as far as we can judge not using administrative register data using population data as we do here. In the empirical studies so far cross-sectional or longitudinal survey data have been used. Health selection to employment, a mechanism where health is an important determinant for both gaining and maintaining employment, appears established empirically, with for instance British, Australian, Swedish, Dutch, and cross-European survey data demonstrating associations and causal relationships between physical and mental health and employment outcomes such as unemployment, disability pension, and early retirement [7–12]. Furthermore, both single-country [13–16] and cross-country [17, 18] studies have found that education influenced the association between chronic illness and employment. Education may for one moderate the employment consequences of chronic illness, i.e., make them less severe for the higher educated, by for instance contributing to sorting workers into specific occupations or by being correlated with higher knowledge about how to cope with one’s chronic disease. Second, the association between health and employment may be mediated by education if, say, the higher educated strata are characterized by both a lower prevalence of chronic disease and a higher employment rate.

In this article, we consider health selection to be a description of a structural process in the Norwegian labour market, and through statistical modelling we attempt to quantify the moderating – or shielding – effect of education, and how this effect changes over time. We do not make a distinction between selection into and out of employment, e.g., by following the labour market trajectories of each individual person, nor do we attempt to estimate the effect of the onset of a chronic disease on employment. Rather, we aim to describe cross-sectional differences between groups defined by health and education status. The causes of these group differences may be located at both the employer and employee level, as well as in the educational or health care system. This article is descriptive in its nature and is not designed to identify these causes.

Further investigation of the consequences of chronic illness is warranted focusing on changes in labour market participation over time. The current study aims to analyse the extent to which the highly educated chronically ill are “shielded” against adverse effects of chronic diseases. We expect to find that the absolute levels of the chronically ill`s labour market participation are likely to be consistently different depending on their level of education. However, we are particularly interested in analysing the magnitude and changes in the differential probability of employment over time. Using a randomly selected 10% sample of the healthy population^3^ as well as non-chronic patients as comparison groups, we study the differences in employment probabilities over time and to what extent level of education influence the “chronic illness penalty”. Finally, chronic conditions are multifaceted, e.g., mental health conditions versus physical health conditions, with potentially different implications for maintaining employment. Physically demanding jobs are likely to be challenging for those with cardiovascular heart diseases and chronic respiratory diseases while office work may be challenging for those that have experienced stroke, cancer treatment or are mentally ill. In the disease specific analyses too, we also study whether the level of education makes a difference compared to a sample of healthy people.

## Data and methods

The study is based on Norwegian administrative data which allows us to combine health data with socio-economic status (SES) data, i.e., level of education. The dataset is obtained from three registry sources: the Norwegian Patient Register (NPR), Statistics Norway (SSB), and the Local Government Dataset [19]. The merged dataset covers more than a decade (2008–2018). For the comparison/reference group of healthy individuals, we consider all individuals aged between 20 and 60 years who have not used specialist health care during our study period. Due to the large number of observations (more than 24 million) that cause numerical overflow, to overcome the computational challenges, we randomly selected a 10% sample of the healthy population by year as one of our reference category. The study population consists of 2,443, 636 observations.

For the two patient groups—chronic and non-chronic– the primary inclusion criterion is hospitalization, i.e., an index hospital episode is defined by the first inpatient treatment during the calendar year. We then separate hospital admission for any of six broad chronic conditions (see the definition below) and pool all other index episodes as non-chronically ill admissions. An additional inclusion criterion, and proxy of severity, is that length of stay is at least one day (LOS > = 1). Lastly, we consider inpatient admissions of people in working age 24–60 years of age. With these inclusion criteria, the study population (chronic and non-chronic patients) consists of 2,169,617 observations of 1,248,978 patients.

### Outcome

Our outcome measure considers the labour market participation on the extensive margin, where employment/labour market participation is defined as whether an individual earned a positive wage income during a year or not (0 = no; 1 = yes).

### Covariates

#### Chronic conditions

According to the WHO (2005) [20] chronic diseases are those that develop and are experienced over prolonged periods of time. The chronic diseases can be influenced by both environmental and individual factors and could be resulted from both modifiable and non-modifiable risk factors. The main chronic diseases are diabetes, cardiovascular disease and stroke, cancer, and chronic respiratory diseases. We identified 15 common chronic conditions according to the validated algorithms for ICD-10 coding of 30 morbidities in administrative data suggested by Tonelli and Colanguages [21]^4^. We categorized 15 common chronic conditions into the following six groups: cancer (lymphoma, metastatic and non-metastatic cancers); cardiovascular diseases (atrial fibrillation, chronic heart failure, hypertension, myocardial infarction and stroke); diabetes; mental health issues (depression and schizophrenia); musculoskeletal conditions (chronic pain and rheumatoid arthritis); and respiratory conditions (asthma and chronic obstructive pulmonary disease). Inpatients admitted with a non-chronic diagnosis constitute the reference category.

#### Education

We classify education into the following three levels (based on the International Standard Classification of Education, ISCED):

- *Low-educated individuals*–those with no education, or with pre-school, primary or lower secondary education, i.e., with 0–10 years of schooling, and those who do not specify their education (omitted category in analyses).
- *Medium-educated individuals*–those who completed upper secondary basic or final year education, or post-secondary non-tertiary education (11–14 years of schooling).
- *Highly educated individuals*- those who completed undergraduate and/or postgraduate tertiary education (15 or more years of schooling).

#### Gender

A categorical variable, for which male = 1 and female = 0.

#### Age

Age is categorized into seven groups, with the youngest group (24–30-year-old) being the reference category.

#### Marital status

Marital status is categorised into three groups: married, unmarried, either divorced or separated or others, and with married considered as a reference category.

#### Immigration status

Immigration status is categorized into 3 groups, native Norwegian and/or born in other Nordic countries, born in Europe or Northern America or Australia or New Zealand, and whether born in Asia, Africa or south or middle America, with the native-born Norwegian and Nordic being the reference category.

#### Labour market regional-level unemployment rates

In 2000, Statistics Norway established a labour market region identifier in Norway based on information on commuting flows [23]. To account for the local labour market condition at the level of the labour market region, we control the unemployment rate (which may indicate the supply and demand situation in each labour market region), defined as the number of registered unemployed persons as a share of the total number of inhabitants aged 16–66 years at the beginning of the year in a labour market region.

### Descriptive statistics

Table 1 shows the composition of healthy people and patients with chronic illnesses who are currently employed. It shows that individuals with high/tertiary level education have the *highest* employment percentage both for the healthy ones and the chronically ill patients (93% vs. 87%), while those with low/primary level education have the lowest, 73% and 55%), respectively.

Regarding the distribution of specific chronic conditions, the relative shares vary between educational level.The lower panel of Table 1 suggests that cancer is the largest category of patients for those with high/tertiary level of education (24%), while those with low/primary level education have the highest shares of patients with, diabetes and respiratory conditions.

The share of low and medium educated patients are for most diagnoses the same. Minor differences between all three educational levels are present for mental health only. However, in absolute numbers the high educated constitutes the smallest groups of patients regardless of diagnoses, while the medium educated group are largest in number of patients with cancer, cardiovascular diseases, diabetes and musculoskeletal conditions. The lower educatedare largest in numbers among those with mentall illness and repiratory conditions.

**Table 1:** Individuals and labour market region composition by health and education levels

Variable	Healthy workers [10% of all healthy population age between 24–60] (*N* = 2, 443, 636)			Chronically ill workers [workers with 15 common chronic conditions and age between 24–60] (*N* = 361,055)		
	Lower Educated (*n* = 502,452)	Medium Educated (*n* = 981,739)	Higher Educated (*n* = 959,445)	Lower Educated (*n* = 110,716)	Medium Educated (*n* = 158,637)	Higher Educated (*n* = 91,702)
	Percentage/ Mean (Std. Dev.)	Percentage/ Mean (Std. Dev.)	Percentage/ Mean (Std. Dev.)	Percentage/ Mean (Std. Dev.)	Percentage/ Mean (Std. Dev.)	Percentage/ Mean (Std. Dev.)
Employment rate	**72.9(44.5)**	**88.6(31.8)**	**92.9(25.7)**	**55.0(49.7)**	**76.2(42.5)**	**86.9(33.8)**
Male	54.2 (49.8)	58.2 (49.3)	44.8 (49.7)	51.0 (50.0)	55.5 (49.7)	41.2 (49.2)
Age (in years)	41.7 (10.8)	43.0 (10.4)	40.6 (10.1)	47.9 (9.52)	48.9 (8.95)	47.2 (9.35)
Married	38.5 (48.7)	45.5 (49.8)	46.6 (49.9)	40.0 (50.0)	49.4 (50.0)	53.7 (49.9)
Unmarried	44.4 (49.7)	40.3 (49.1)	42.4 (49.4)	34.0 (47.4)	28.6 (45.2)	28.8 (45.2)
Divorced/separated/others	15.3 (36.0)	13.4 (34.1)	09.4 (29.2)	25.7 (43.7)	21.8 (41.3)	17.3 (37.8)
Native Norwegian & Nordic	75.3 (43.1)	88.4 (32.0)	84.5 (36.2)	86.5 (34.2)	92.1 (26.9)	87.8 (32.7)
Europe/North America/Australia	8.60 (28.0)	7.20 (25.8)	9.10 (28.7)	4.13 (19.9)	4.13 (19.9)	6.10 (23.9)
Asia/Africa/South America	16.1 (36.8)	04.4 (20.5)	6.40 (24.5)	9.39 (29.2)	3.72 (18.9)	6.01 (23.9)
Labour market regional unemployment	2.09 (0.54)	2.05 (0.55)	2.09 (0.55)	2.11 (0.52)	2.07 (0.53)	2.10 (0.53)
**Composition of specific chronic patients**				Lower Educated	Medium Educated	Higher Educated
Cancer				12.3 (32.8)	16.7 (37.3)	23.9 (42.6)
Cardiovascular				27.8 (44.8)	30.0 (45.8)	25.6 (43.7)
Diabetes				8.30 (27.6)	6.10 (24.0)	5.06 (21.9)
Mental illness				02.0 (13.9)	1.14 (10.6)	1.09 (10.4)
Musculoskeletal conditions				40.6 (49.1)	40.6 (49.1)	39.8 (48.9)
Respiratory conditions				9.01 (28.7)	5.55 (22.9)	4.50 (20.8)

**Fig. 1:**
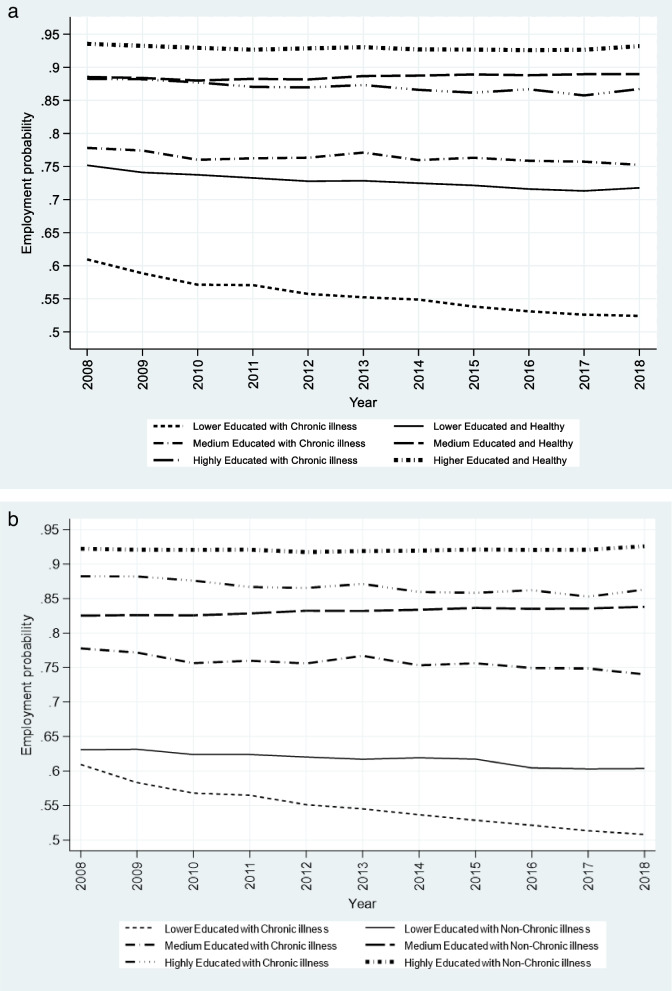
**a** Employment probability between healthy and chronic patients by education (2008–2018). **b** Employment probability all chronic and non-chronic patients by education (2008–2018)

Figure 1a depicts differences in the employment probabilities of chronic patients and healthy people by levels of education over time. The employment probabilities of chronic patients with low level of education seem to deteriorate over time, from a low level of 0.62 in 2008 to 0.52 in 2018, a reduction of 16%. The employment probabilities are also declining for the medium and highly educated chronically ill but less so and from a higher level. We also note that the “raw” data reveals a gradient: The high educated chronic patients have the highest employment probabilities, the medium educated follow and chronically ill individuals with lower education are the less fortunate. The hierarchical structure is also observed among the healthy individuals.

In Fig. 1b we capture the differences in employment probabilities of chronic and non-chronic patients across levels of education over time. The hierarchical structure is also observed among the non-chronically ill (the solid lines). The most notable trend in Fig. 1b is that the employment probabilities of non-chronic patients with low education also seem to deteriorate over time but less so compared to chronic patients with low education. Here we also notice a gradient by education: The high educated non-chronic patients have the highest employment probabilities, and the medium educated follow.

Figure A1 (in Appendix A) shows remarkable differences in employment probabilities depending on diagnoses. While musculoskeletal, cardiovascular and partly cancer patients have similar patterns as all chronic conditions pooled (Fig. 1a), i.e., patients are “sorted” hierarchically according to level of education, patients with diabetes, respiratory and mental illness are not.

#### Statistical analysis

We use linear probability models with labour market region and year fixed effects^5^. To account simultaneously for two sources of unobserved heterogeneity—the year and the labour market region—the number of units become too large. Therefore, we employ the following high-dimensional fixed-effects model by using the Stata module *reghdfe* (Correia, 2014) [24]:

1 $$l_{irt}\;=\;X_{irt}\;\;\beta\;+\;\theta_{rt}\;\delta\;+\;\lambda_r\;+\;\delta_t\;+\;\varepsilon_{irt}$$

where the dependent variable, labour market participation—employment probability— l_irt_, equals one if person *i* belong to labour market region *r* in year *t* has a positive wage income, and zero otherwise. To reduce bias due to patients’ heterogeneity, we included a series of covariates that may influence the association: individual demographic and socioeconomic characteristics as well as chronic health conditions, as detailed above and X_irt_ includes these characteristics of workers. 𝜃_rt_ comprises labour market region characteristic, i.e., unemployment rates. Furthermore, a fixed-effects approach at labour market region, Λr𝜆_r_ can reduce the effect of unmeasured confounding. In the context of this analysis, the labour market regional fixed-effects estimator controls for any time-invariant differences between regions, such as centrality, governance structure or the size of the catchment area which may influence the availability of specialist healthcare services, such as distance to hospital. Since the fixed effects estimator relies only on variation within the chosen unit, this estimator is not affected by confounding from unmeasured time-invariant factors. $\delta$_*t*_ includes period-specific fixed effects common to all patients and all labour market regions, and $\epsilon$_*irt*_ is unexplained random variation.

The models are further extended by including the interaction effects between chronic illness, level of education and period to explore the ‘shielding effect’ of education over time. Equation (1) can be rewritten as:

2 $$l_{irt}=chronic_{irt}\;\ast edu_{irt}\;\ast time\;\beta{'}\;+\;D_{irt}\;\beta{"}+\;\theta_{rt}\;\delta\;+\;\:\lambda_r\:+\:\varepsilon_{irt'}$$

where *D*_*irt*_ includes all individual characteristics as mentioned above except chronic health condition and education. Since our outcome is binary, non-linear models such as logit or probit models may seem appropriate. However, estimating a fixed-effect logit model resulted in numerical overflow and the interpretation of the interaction terms are not straightforward. Therefore, we have chosen to estimate linear fixed-effect models rather than non-linear models without fixed effect. We cluster the standard errors at the level of the labour market region in all specifications.

## Results

The multivariate regression estimates using Eq. 1 are reported in Table 2. People hospitalized with a chronic condition have on average approximately 11% lower probability of being in the labour market compared to the healthy (non-hospitalized) individuals (Column 1). The employment probability for the chronically ill is 4% lower than their non-chronically ill counterparts (Column 2).

As expected, individuals with lower education have lower employment probabilities compared to the medium and higher educated. Compared to the healthy population, the probability is 15% and 20% lower, respectively (Column 1). Compared to the non-chronically ill (Column 2), the differences are bigger, 20% and 27%, indicating that the chronically ill seems to be faced with substantially different challenges in the labour market compared to other groups of hospitalized patients.

The results from the disease-specific analyses indicate that chronically ill patients have lower probabilities of employment relative to the healthy population (Column 3–8), as expected. We notice that patients diagnosed with mental illness have the lowest employment probability compared to the healthy ones (33%, Column 6). The smallest difference is for cancer patients with a 5% lower employment probability compared to the healthy population (Column 3).

We find it striking that the “shielding effect” of education is so pronounced even for the specific diagnoses. The medium educated chronically ill, regardless of disease, have on average around 14% higher probability of employment than the lower educated (Column 3–8), while the higher educated have on average 18% higher probability of employment.

**Table 2:** Labour market participate estimates for all chronic conditions and condition-specific illnesses

Variables	(1) All chronic vs. healthy	(2) All chronic vs. Non-chronic	(3) Cancer vs. healthy	(4) Cardiovascular vs. healthy	(5) Diabetes vs. healthy	(6) Mental ill health vs. healthy	(7) Musculoskeletal vs. healthy	(8) Respiratory vs. Healthy
Chronic	-0.107^***^	-0.039^***^	-0.046^***^	-0.088^***^	-0.185^***^	-0.332^***^	-0.108^***^	-0.271^***^
condition	[-0.111,-0.103]	[-0.0,043,-0.0,035]	[-0.052,-0.040]	[-0.092,-0.084]	[-0.193,-0.176]	[-0.358,-0.306]	[-0.113,-0.102]	[-0.282,-0.259]
Medium	0.148^***^	0.196^***^	0.138^***^	0.140^***^	0.138^***^	0.137^***^	0.141^***^	0.139^***^
Education	[0.142,0.154]	[0.188,0.204]	[0.132,0.145]	[0.134,0.146]	[0.132,0.145]	[0.131,0.144]	[0.135,0.147]	[0.132,0.145]
High	0.196^***^	0.272***	0.181^***^	0.183^***^	0.181^***^	0.180^***^	0.186^***^	0.182^***^
Education	[0.188,0.204]	[0.262,0.282]	[0.173,0.190]	[0.175,0.192]	[0.173,0.189]	[0.171,0.188]	[0.178,0.194]	[0.173,0.190]
N	2,804,691	2,169,617	2,505,561	2,545,390	2,467,213	2,448,624	2,589,475	2,466,608
Adj. R^2^	0.097	0.131	0.077	0.0812	0.080	0.078	0.085	0.085

95% confidence intervals in brackets

^*^p < 0.05, ^**^p < 0.01, ^***^p < 0.001

All models are also controlled for gender, age groups, marital and immigration status as well as year and labour market region fixed effects. Detailed regression results for the two alternative comparison groups are reported in Table A1 in the Appendix.

**Fig. 2:**
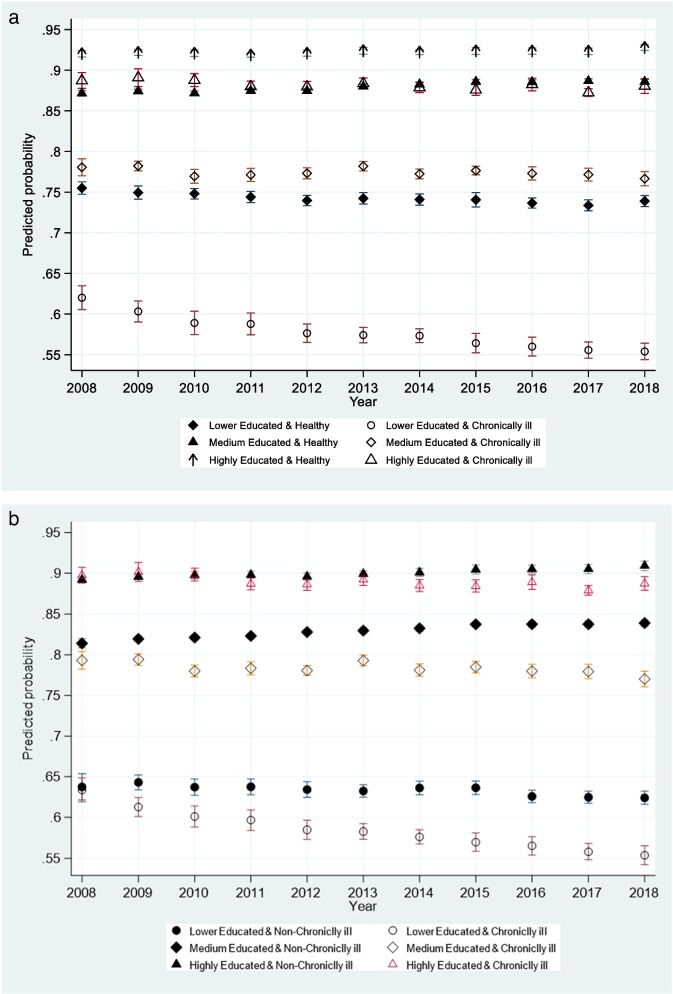
** a **illustrates the gradient by education in predicted employment probabilities for healthy individuals, too. Nevertheless, irrespective of the level of education of the healthy individuals, the predicted probabilities look constant over the 11 years period or even slightly increasing for those with higher education. **b** Predicted probability of employment (with 95% CI), between healthy individuals, chronically ill patients, and level of education, over the years

 Using Eq. 2, the predicted employment probabilities (with 95% confidence interval) are depicted in Fig. 2a. While it is not surprising that education seem to “shield” against unfortunate labour market consequences - in the sense that the employment probability is highest for the high educated and lowest for chronically ill individuals with lower education - what is revealing is the trends, the extent to which the differences between the educational groups are changing over time. The mean predicted employment probability for the high educated chronic patient changes from 89% in 2008 to 88% in 2018, whereas the predicted employment probability changes from 62% in 2008 to 55% in 2018 for chronic patient with lower education.

The predicted employment probabilities between the non-chronically ill patients and chronically ill patients are shown in Fig. 2b. The predicted employment probabilities of non-chronically ill across levels of education are significant and increasing over time for both the high, medium, and low-educated groups using 2008 as the baseline. Focusing on the chronically ill groups, the figure reveals a pattern. The differential probabilities between chronically ill and non-chronically have increased over time for the three educational groups. However, the differential probability of employment is the smallest among the highly educated and largest among the low-educated comparing 2008 and 2018. Noting that the confidence intervals overlap throughout most of the period, the change over time for the high educated chronically ill is not significant. The non-significant changes over the 11-year period implies that the high educated as a group seems to be able to maintain the labour market participation as we define it here. In contrast, the medium level educated chronically ill patients seem to experience a significant negative trend. The reduction in participation rates comparing the levels of 2008 (79%) and 2018 (77%) is 2.5%. Still, the most revealing development is found among the lower-educated chronically ill. The trend is negative and significant showing a reduction in participation rates of 12.7% over the 11-year time period (63% and 55%).

These results suggest that the highly educated are “shielded” not only in terms of absolute levels of employment probabilities (i.e., non-significant change over time) but also relative to their non-chronically ill comparison group. Admittedly, from 2014 onwards, the differential probability is positive and significant for the medium and lower educated only. Nevertheless, the highly educated as a group is substantially more shielded

The disease-specific analyses give a more complex picture compared to the pooled analyses (see Figure A2 in Appendix A). The low-educated with diabetes, musculoskeletal diseases and respiratory diseases all have a significant reduction in employment probabilities comparing the endpoints of the period 2008–2018. While diabetes, musculoskeletal diseases and respiratory diseases may reflect lifestyle factors more prevalent among the low-educated, it is interesting to find that employment seem to be more difficult to hold on too. 

For mental health, individuals with lower education have the lowest employment probabilities of all conditions with a decreasing mean (not significant change over time, though), but we find no differences between the medium and highly educated. This indicates that the high-educated with mental illness are not shielded to the extent that the results from the pooled data suggest. The result may reflect that the high-educated have tasks at work that somehow becomes too complicated or demands more diligence and presence at work than the disease allow.

## Discussion

The main aim of this article was to explore whether labour market participation is unequally shared across socioeconomic strata and, whether the differences in labour market participation between the high, medium and low-educated chronically ill have changed over time. Emphasizing that we have not evaluated the effects of any specific policy measure, what we do know is that throughout the period different policies have been promoted to increase the labour market participation rates of the chronically ill in Norway. In terms of the effect of the Inclusive Working Life (IWL) agreement and other efforts made by local and central government agencies to keep the chronically ill in the labour force, our results may raise some concerns. Based on our findings, one potential concern is that population-level policy measures may make a difference for the highly educated and less so for the lower educated groups of patients keeping in mind that we have controlled for changes in unemployment rates over time in our analyses.

Several mechanisms could explain the education-related SES inequalities in employment among the chronically ill, which concern both employer and employee perspectives and describe selections out of and into employment. First, working life demands may differ between the jobs available for the lower and higher educated, leading to a higher degree of selection *out of* employment for chronically ill individuals with lower education. This explanation implies treating education as a proxy for occupational status and, subsequently, as a predictor of working conditions. Does it reflect that physically demanding jobs have become even more of a challenge over time? Or that employers are less willing to accommodate needs for adjustments in job tasks and/or working hours? There may be more exposure to health-damaging risks in ‘low-education jobs’ that are difficult for a person with a chronic disease to endure, such as heavy lifting, vibration from tools or machinery, extreme temperatures or noise and air pollution (d’Errico et al., 2021) [25]. In addition, as the Covid-19 pandemic has shown, the need to be present at work is not equal across the segments of the labour market and not being able or permitted to work from home may increase an individual’s risk of virus exposure (cf. Lyttelton & Zang, 2022) [26]. Moreover, working from home may provide an employee with a chronic disease with the opportunity to rest and recover more easily than being present at work.

The chronically ill may also differ in terms of their intrinsic motivation to stay in the labour market. The issue of potential self-selection out of the labour market is relevant in a Norwegian context. The Norwegian welfare state is characterized by generous benefits, such that an employee’s total income is likely to remain stable despite any deterioration in health. This suggests that for people with lower expected productivity, such as those with chronic health problems, and/or low education, their marginal probability of being in the labour market could be small. In addition, most benefits (including unemployment and sickness) are capped at six times the so-called “Basic amount” (6G) in the national insurance scheme (6G corresponding to NOK 421 536 in 2008 and NOK 581 298 in 2018). This gives the highest earners – which are likely to be more prevalent in the highest educated group – a greater incentive to return to work (Vaalavuo, 2021) [27].

Education-related inequality in employment may imply that the higher educated are less affected by their registered illness. They may have other strategies and resources available to manage their disease and cope with the demands of working life, such as beneficial knowledge of available treatments and ways to relieve symptoms, health behaviours and self-management strategies. It could be assumed that the hospitalized inpatients whom we study are equally affected by their diseases, given that all required hospital admission. However, there is a multitude of reasons for admission due to chronic illness (e.g., surgery, screenings and routine check-ups); it is possible that the reasons are heterogeneous across education categories and therefore, influence our results.

To sum up, our results point in the direction that the highly educated chronically ill are more “shielded” both in relative and absolute terms against unfortunate consequences of their illness compared to the other two groups. Out of the six categories of chronically ill patients, mental health diagnoses have the lowest probability of labour market participation, irrespective of education level. For this group of patients, we do not find that education has a shielding effect, which suggests that those with the included diagnoses (depression and schizophrenia) find it particularly challenging to combine their chronic illness with the demands of working life, regardless of occupation.

Importantly, policy measures may have contributed to mediate the impact of chronic illness, i.e., the negative trends observed in our data could have been worse without them. Still, the trends for the chronically ill individuals with lower education need attention from policymakers. The increasing differences in participation rates point towards increased inequality, with ramification for future differences in levels of pensions and living standards in retirement.

Our study complements previously reported studies from Norway [14, 15], either by using more recent data or register data rather than survey data. In terms of labour market participation, our register data analyses illuminate the relevance of studying the associations related to health status and level of education. One of the main strengths of our study is that we utilized rich register datasets that cover a decade, which enable us to diminish diverse types of biases. Much of the previous research has used survey data and self-reported chronic disease and employment data. Therefore, other related studies may be prone to *reporting bias* or suffer from *misclassification bias* in defining different chronic conditions. Studies using survey data may be problematic for countries with small populations, especially in Norway, where a small portion of the working-age population is unemployed and, therefore, there may be small samples for meaningful inferences. Moreover, our data enable us to differentiate our results by diagnosis. Another strength is our sample selection because we consider patients with both chronic and non-chronic conditions and compare labour market participation. Therefore, it is likely that our results are not affected by differences in the definition or perception of poor health because all patients have sufficiently serious health problems in that they have been admitted to hospital during the study period. Furthermore, it is unlikely that our findings are affected by SES inequalities in the use of health services because all patients included in our study utilised specialist health services during the study period. We also compare the chronically ill with a sample from the healthy population adding relevance of our findings.

There are some limitations connected with our study. If one considers that health selection is stronger when demand for labour is low, individuals pre-hospitalization situation in the labour market is a potentially important factor. We only capture whether the individual has received labour income the same year as hospitalization. However, we do control for regional unemployment rates in the analyses. Moreover, we do not differentiate between industries, and it is conceivable the type of industry an individual is employed in has bearing on the labour market possibilities across all levels of education.

Furthermore, causal inference is difficult. Estimation of the effects of health and education is complicated by possible endogeneity bias owing to unobserved characteristics of patients, which may influence both health and education as well as the decision to be in the labour market. Owing to the simultaneous determination of chronic ill health and employment prospects, one cannot rule out biases due to reverse causality.

## Conclusions

In Norway, a country characterized by high overall employment, inclusive working life and active labour market policies, the employment prospects for people with chronic health conditions nevertheless remain an important issue on the political agenda, e.g., [28, 29]. Our study suggests that both chronic diseases and the impact of chronic diseases are unequally shared across socio-economic groups. We concluded that education - as an indicator of SES - functions as a shield to minimize the negative effects of chronic illnesses on employment. On average, a chronically ill person with a higher level of education has a higher employment probability than patients with a lower level of education. There are differences across the chronic conditions in the employment probability, though, indicating different magnitudes of the shielding effect of education. In particular, workers with mental illness face barriers in the labour market regardless of educational qualifications.

In the Scandinavian context, recent systematic review findings suggest that research on the work inclusion of people with chronic illnesses is a field with a small evidence base, wherein several interventions concentrate on the “treatment” of specific chronic condition groups [30]. Most parts of the evidence describe supply-side interventions. For example, promising trials with individual placement and support methods have been identified for those with chronic mental illness [31]. The findings also suggest that there is a need for efforts not only on the supply side of the labour market (e.g., through improving the employability of marginalized groups) but also on the demand side to require and/or incentivize employers to hire and retain employees with lower expected productivity [32, 33, 34]. To the extent that chronically ill individuals with lower education are employed in enterprises that find it difficult to facilitate working conditions and/or are doing tasks that are unfavourable given their disease, it is likely that both the carrot and the stick should be used to prolong the chronically ills´ participation in the labour market.

## Supplementary Information

**Supplementary Material 1.**

## Data Availability

The data analyses are based on administrative register data stored on a safe server at University of Bergen, Norway. The data are available for a limited group of researchers only and can not be shared publicly.

## Footnotes

1 IWL was last extended in December 2018 https://www.regjeringen.no/contentassets/fc3b4fed90b146499b90947491c846ad/the-ia-agreement-20192022.pdf.

2 See for instance https://www.eurofound.europa.eu/sr/publications/report/2014/norway-employment-opportunities-for-people-with-chronic-diseases)

3 Healthy in the sense that those who make up the samples has not been hospitalized during a given year. In this study, we have not been able to identify those that have used primary health care, implying that the yearly samples may consist of individuals with and without health issues.

4 The study identified validated algorithms for ICD-10 coding of 30 morbidities in administrative data. These algorithms are also used by previous studies, see for example, Stokes et al. (2021) [22].

5 Our individual-level data is highly imbalance as most of the individuals are only observed one to two times (waves). Dropping singleton observations may risk introducing an appreciable bias into the sample which would likely affect the results. Therefore, we use pooled individual-level data with labour market region and time-fixed effects approach but not individual-level fixed-effect.
