# Supplementary material for: The chronically ill in the labour market – are they hierarchically sorted by education?
Source: Int J Equity Health. 2024 Mar 25;23:66. doi: 10.1186/s12939-024-02148-w (PMC11409748; doi:10.1186/s12939-024-02148-w)

Table A1: Detailed regression results for two alternative comparison groups

|  | **Healthy versus Chronic patients** | | **Non-chronic versus Chronic patients** | |
| --- | --- | --- | --- | --- |
| Coefficient | Coefficient | 95% Confidence Interval | Coefficient | 95% Confidence Interval |
| **Chronic illness** | **-0.107** | **-0.111, -0.103** | **-0.039** | **-0.043, -0.036** |
| Education (Low education reference category) | | | | |
| Medium education | 0.148 | 0.142, 0.154 | 0.196 | 0.188, 0.204 |
| High education | 0.196 | 0.188, 0.204 | 0.272 | 0.262, 0.282 |
| male | 0.027 | 0.024, 0.029 | 0.029 | 0.023, 0.035 |
| Marital status (married reference category) | | | | |
| Unmarried | -0.053 | -0.057, -0.048 | -0.064 | -0.069, -0.059 |
| Divorced/separated/others | -0.044 | -0.048, -0.041 | -0.068 | -0.072, -0.064 |
| Immigration status (Native Norwegian/Nordic reference category) | | | | |
| Europe/North America/Australia | -0.057 | -0.066, -0.048 | -0.051 | -0.059, -0.044 |
| Asia/Africa/South America | -0.158 | -0.163, -0.154 | -0.177 | -0.186, -0.168 |
| Labour market regional unemployment | -0.005 | -0.007, -0.003 | -0.005 | -0.008, -0.002 |
| Age group (age 24–30-year reference category) | | | | |
| Age 31-35 | -0.017 | -0.020, -0.014 | -0.024 | -0.032, -0.015 |
| Age 36-40 | -0.031 | -0.034, -0.027 | -0.055 | -0.066, -0.045 |
| Age 41-45 | -0.043 | -0.049, -0.038 | -0.089 | -0.094, -0.083 |
| Age 36-50 | -0.055 | -0.063, -0.047 | -0.108 | -0.114, -0.102 |
| Age 51-55 | -0.078 | -0.087, -0.069 | -0.137 | -0.145, -0.129 |
| Age 56--60 | -0.126 | -0.135, -0.117 | -0.190 | -0.198, -0.182 |
| Year (2008 reference category) | | | | |
| 2009 | 0.002 | 0.000, 0.005 | 0.004 | -0.001, 0.009 |
| 2010 | 0.000 | -0.002, 0.002 | 0.003 | -0.002, 0.008 |
| 2011 | -0.002 | -0.004, 0.000 | 0.004 | -0.002, 0.009 |
| 2012 | -0.002 | -0.004, 0.001 | 0.003 | -0.002, 0.008 |
| 2013 | 0.002 | -0.001, 0.005 | 0.005 | -0.001, 0.011 |
| 2014 | 0.002 | -0.001, 0.005 | 0.006 | -0.001, 0.013 |
| 2015 | 0.004 | 0.000, 0.007 | 0.009 | 0.000, 0.017 |
| 2016 | 0.002 | -0.002, 0.006 | 0.006 | -0.003, 0.015 |
| 2017 | 0.001 | -0.002, 0.004 | 0.005 | -0.003, 0.013 |
| 2018 | 0.003 | 0.000, 0.006 | 0.006 | -0.002, 0.015 |
| Adjusted R^2^  Number of Observation | | 0.097  2.804.691 |  | 0.1312  2.169.617 |

Note: All models are also controlled for labour market region fixed effects with robust standard errors

Figure A1: Labour market participation differences between specific chronic conditions and non-chronic patients by education levels, 2008–2018

**Cancer**


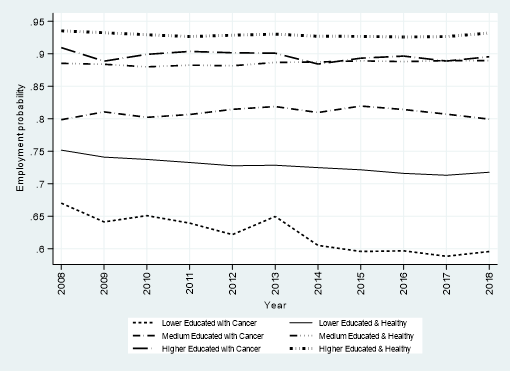


**Cardiovascular**

 
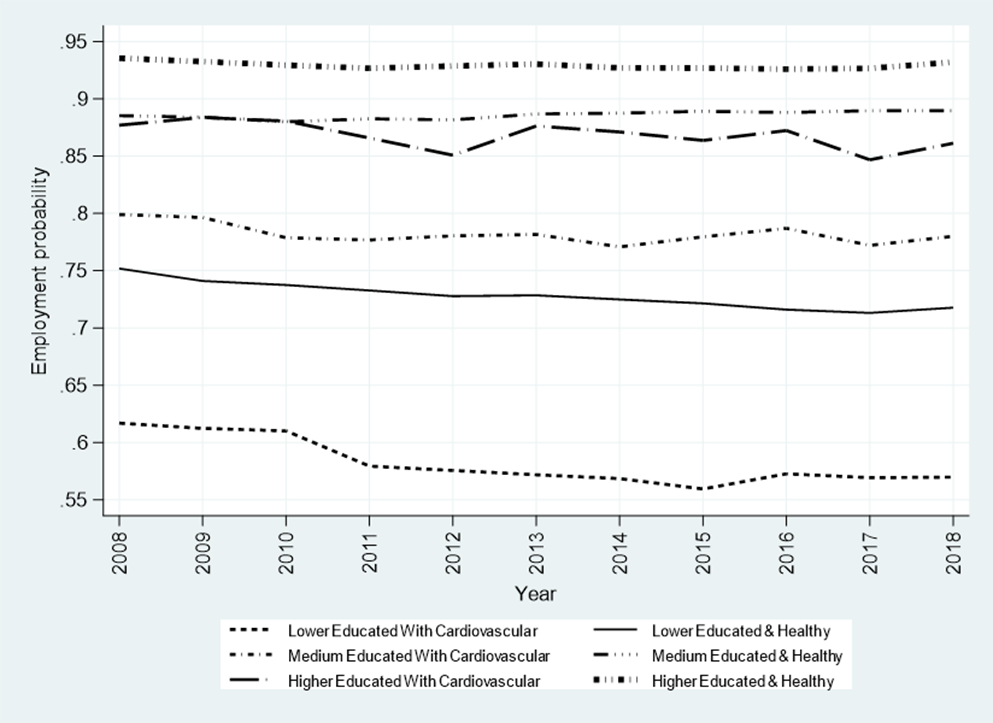


**Diabetes**


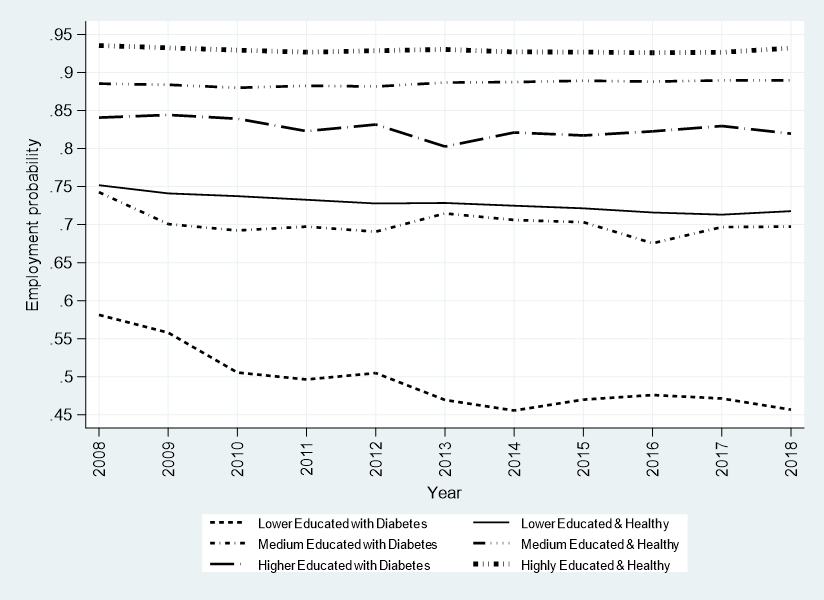


**Mental illness**


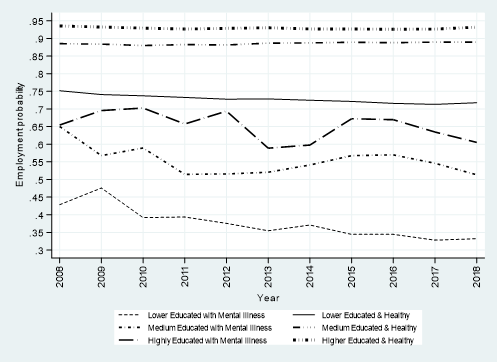


**Musculoskeletal illness**


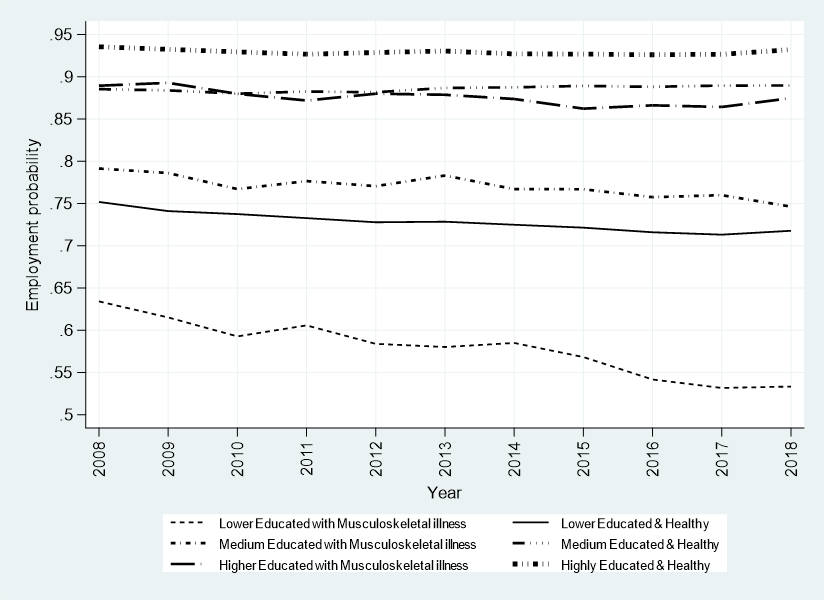


**Respiratory illness**


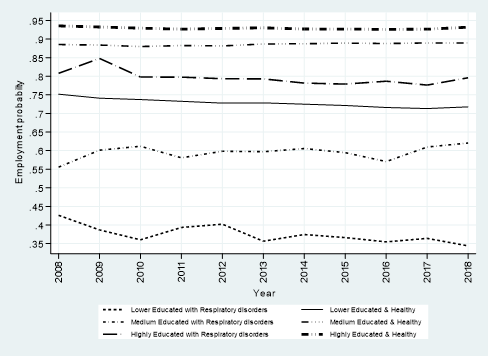
 Figure A2: Predictive probabilities of being employed by education levels and specific chronic illness status over the years 2008-2018

**Healthy Versus Cancer**
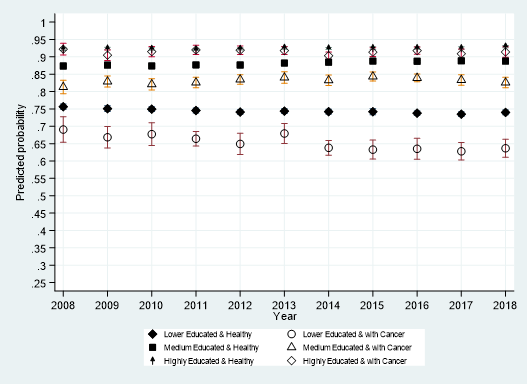


**Healthy Versus Cardiovascular**


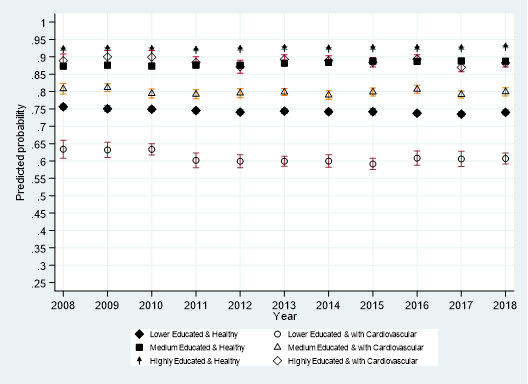


**Healthy Versus Diabetes**


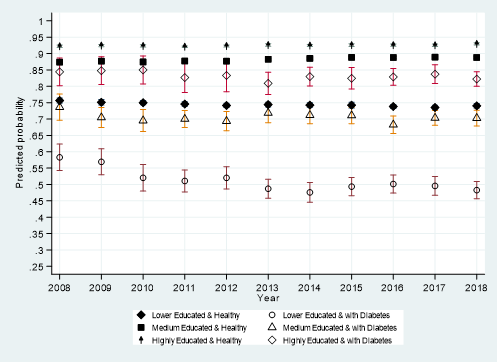


**Healthy Versus Mental ill health**


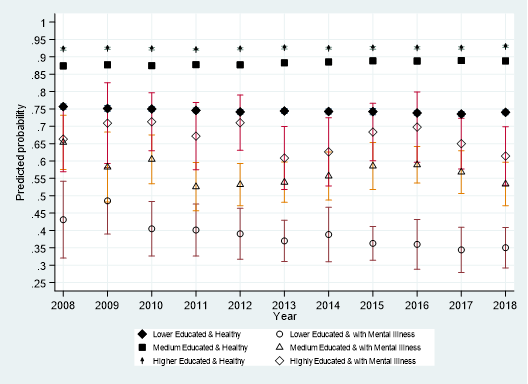


**Healthy Versus Musculoskeletal**


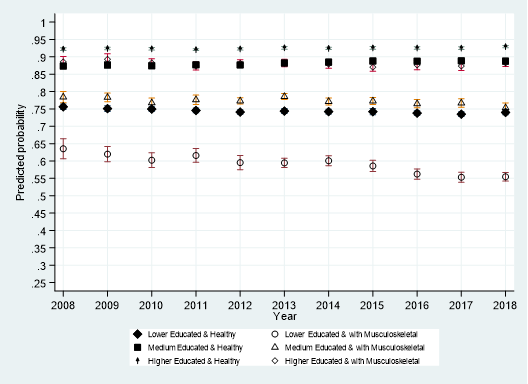


**Health Versus Respiratory**


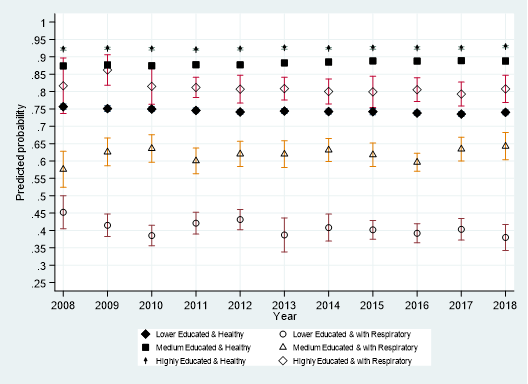

Supplement: Supplementary file 1 — Supplementary Material 1. [file 12939_2024_2148_MOESM1_ESM.docx]
